# Novel Techniques to Unravel Causative Bacterial Ecological Shifts in Chronic Urinary Tract Infection

**DOI:** 10.3390/pathogens14030299

**Published:** 2025-03-20

**Authors:** Catherine C. Y. Chieng, Qingyang Kong, Natasha S. Y. Liou, Mariña Neira Rey, Katie L. Dalby, Neil Jones, Rajvinder Khasriya, Harry Horsley

**Affiliations:** 1 Centre for Kidney and Bladder Health, University College London, London NW3 2PF, UK; c.chieng@ucl.ac.uk (C.C.Y.C.); natasha.liou.14@ucl.ac.uk (N.S.Y.L.); marina.rey.23@ucl.ac.uk (M.N.R.); katie.dalby.24@ucl.ac.uk (K.L.D.); 2 Department of Microbial Diseases, Eastman Dental Institute, University College London, London NW3 2PF, UK; qingyang.kong@ucl.ac.uk (Q.K.); r.khasriya@ucl.ac.uk (R.K.); 3 EGA Institute for Women’s Health, University College London, London WC1E 6AU, UK; 4 Microbiology Department, Whittington Health NHS Trust, London N19 5NF, UK; neil.jones6@nhs.net

**Keywords:** urinary tract infection, lower urinary tract symptoms, pain, host immune response, urinary leukocytes, chromogenic agar culture, 16S rRNA sequencing, K-means clustering, bladder pain syndrome, painful bladder syndrome

## Abstract

Chronic urinary tract infection (UTI) presents with protracted lower urinary tract symptoms and elevated urinary leukocyte counts, but its bacterial etiological agents remain obscure. In this cross-sectional investigation, we aimed to unravel the role of the bladder microbiota in chronic UTI pathogenesis by studying the host immune response. Urine samples were collected from healthy controls (HT), chronic UTI patients who had not initiated treatment (PT) and those undergoing treatment (OT), then sorted into white blood cell (WBC) and epithelial cell (EPC) fractions. Bacteria associated with both fractions were identified by chromogenic agar culture coupled with mass spectrometry and 16S rRNA sequencing. Distinct WBC-exclusive bacteria were observed in the healthy population, but this pattern was less obvious in patients, plausibly due to epithelial shedding and breaching of the urothelial barrier. We also described a bacterial fingerprint guided by *Escherichia* that was able to stratify patients based on symptom severity. Clustering analyses of mean rank changes revealed highly statistically significant upward and downward ecological shifts in communities of bacteria between the healthy and diseased populations. Interestingly, many of the most abundant genera identified in sequencing remained stable when compared between the study cohorts. We concluded that reshuffling of the urinary microbiome, rather than the activity of a single known urinary pathogen, could drive chronic UTI.

## 1. Introduction

Urinary tract infection (UTI) preponderantly affects females, with an age-standardized incidence rate of 80 per 1000—nearly four times that in males. While the global age-standardized disability-adjusted life-year (DALY) rate declined from 1990 to 2019, it has remained largely unchanged during the same period for UTI, reflecting a persistent health burden [1,2]. Approximately 26% of UTI cases recur once within a year [3], with some patients experiencing chronic lower urinary tract symptoms (LUTS) over several years [4].

Standard diagnostics, including the biochemical urinary dipstick and midstream urine (MSU) culture, often lack sensitivity and specificity, leading to delayed or inaccurate UTI treatment [5,6,7,8]. The presence of epithelial cells is traditionally considered as contamination and renders the urine sample unusable, hence the recommendation for a clean-catch MSU sample. Modern studies, however, have shown that majority of the epithelial cells found in the urine originate from the urinary tract [9,10] and urine collection methods have no effect on the incidence of bacterial contamination [11]. Instead, elevated counts of infected epithelial cells and white blood cells, together with voiding and pain symptoms, are key markers of chronic UTI [4,10,12,13,14,15,16].

Multiple independent studies have confirmed the presence of resident bladder microbiota through methods such as 16S rRNA metagenomic sequencing and enhanced culture techniques [17,18,19,20], complicating the distinction between commensals and pathogens. Most urobiome studies focus on distinguishing bacterial profiles in the healthy population versus cohorts with defined LUTS, revealing putative health- or disease-associated bacteria, but sample heterogeneity limits conclusions [21,22,23,24,25,26,27,28]. Similarly, studies comparing the urobiome between chronic UTI patients and healthy controls have thus far been unable to determine definitive differences [7,19]. Indicators of microbiome dysbiosis, such as reduced diversity [29] and imbalances in relative abundance ratios [30], have been postulated as potential drivers of disease in UTI.

We aimed to explore the relationship between the resident bacterial ecology and bacteria responsible for eliciting an immune response in the bladder using shed urinary epithelial cell-associated and urinary white blood cell-associated bacteria as respective proxies. To achieve this, we sorted urinary cells harvested from chronic UTI patients and age-matched healthy controls into white blood cell (WBC) and epithelial cell (EPC) fractions and used agar culture and 16S rRNA sequencing to study bacterial populations. By employing novel clustering techniques and analyses, we uncovered potential ecological shifts, microbial fingerprints and polymicrobial infection in the etiology of chronic UTI.

## 2. Materials and Methods

### 2.1. Sample Processing and Sorting into Urinary White Blood Cell and Epithelial Cell Fractions

This study was approved by Health and Care Research Wales (reference: 22/WA/0069). Urine samples as per hospital standard operating procedures were collected with informed consent from female adult outpatients at the Whittington Health and Royal Free Hospitals (London, UK) and categorized into three study groups. The healthy (HT, n = 18) group comprised of individuals not currently diagnosed with UTI and who had not taken antibiotics for at least a month prior to sampling. The chronic UTI pre-treatment (PT, n = 14) group comprised of patients diagnosed with chronic UTI who were new to the LUTS clinic and were not on antibiotic treatment for at least two weeks prior to sampling. The chronic UTI on treatment (OT, n = 19) group comprised of patients diagnosed with chronic UTI who were being treated at the LUTS clinic with antibiotics and/or urinary antiseptics. None of the participants were immunocompromised or suffered from comorbidities that might influence this study. The patients typically experienced UTI symptoms for an average of 6.5 years before first attendance to the specialist tertiary referral LUTS service [4]. All participants provided personal particulars and answered a 39-point symptom questionnaire [13]. Fresh urine microscopy was performed to enumerate epithelial and white blood cells. The samples underwent routine urinalysis (Multistix^®^ 8 SG Reagent Strips, Siemens Healthineers, Surrey, UK) and overnight MSU culture on chromogenic agar (CHROMID^®^ CPS^®^ Elite Agar, CPSE, bioMérieux, Basingstoke, UK) using a 10 μL calibrated loop and standard surface streak technique.

**Figure 1:**
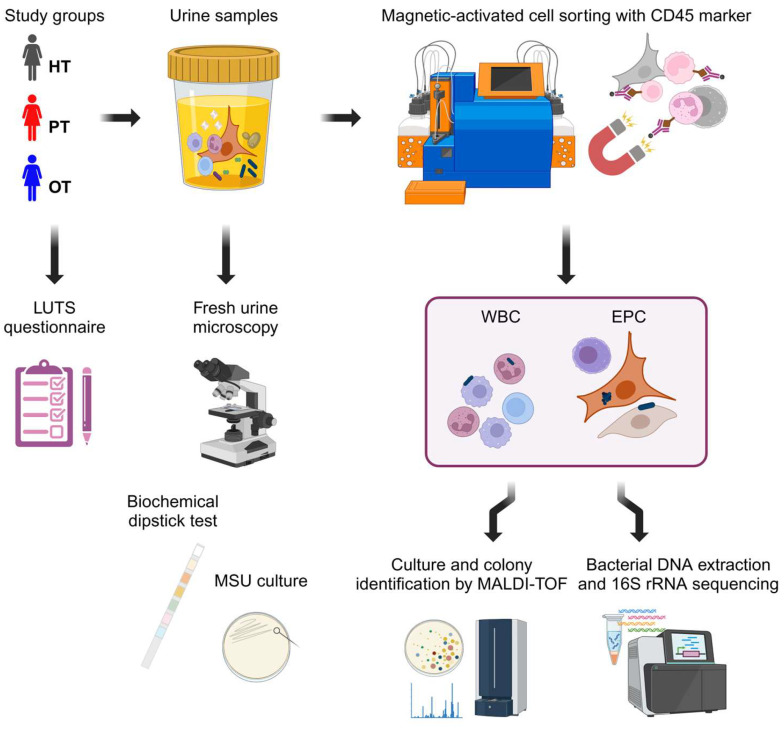
Experimental design. Three study cohorts answered a 39-point LUTS questionnaire and provided urine samples. Fresh urine microscopy was performed to enumerate urinary white blood cells and epithelial cells followed by standard diagnostic tests with biochemical dipstick and MSU culture. The urine samples were then sorted via CD45 magnetic-activated cell sorting into leukocyte-enriched WBC and leukocyte-depleted EPC fractions. These fractions were subsequently cultured and sequenced to examine the bacterial profiles. Figure created in BioRender.com. HT: healthy; PT: chronic UTI pre-treatment; OT: chronic UTI on treatment; LUTS: lower urinary tract symptoms; MSU: midstream urine; WBC: white blood cell fraction; EPC: epithelial cell fraction.

The urine samples were then transported in insulated cool boxes with ice packs to the research laboratory within four hours for immediate processing and storage. They were centrifuged at 300× *g* for 5 min at room temperature and pelleted cells were stored at −70 °C in cryopreservation medium (CTS^TM^ Synth-a-Freeze^TM^ Medium, ThermoFisher, Oxford, UK) until further use within 1 to 10 months. On the day of experimentation, urinary cells were thawed and incubated with anti-human CD45 MicroBeads (Miltenyi Biotec, Surrey, UK) for magnetic-activated cell sorting (autoMACS^®^ Pro Separator, Miltenyi Biotec, Surrey, UK), resulting in leukocyte-enriched white blood cell (WBC) and leukocyte-depleted epithelial cell (EPC) fractions. For every fraction before (NEAT) and after CD45 marker separation (WBC and EPC), 0.1 mL of samples at a concentration of 125,000 cells/mL were cultured overnight at 37 °C on CPSE. Each individual morphological colony type was then sub-cultured on new agar plates to obtain pure colonies for identification by matrix-assisted laser desorption/ionization time-of-flight (MALDI-TOF) mass spectrometry with a score threshold of ≥2.00. The remaining samples from WBC and EPC fractions were stored in DNA/RNA Shield (Zymo Research, Freiburg, Germany) at −70 °C for subsequent DNA extraction and 16s RNA sequencing (Figure 1).

### 2.2. PNA FISH and Immunofluorescence

A randomly selected subset of urine samples (n = 10 per study group) was prepared for fluorescence in situ hybridization using peptide nucleic acid (PNA FISH) and immunofluorescence staining. Cytocentrifugation was conducted using a Shandon Cytospin 2 at 800 rpm for 5 min at room temperature and adhesion slides (Epredia^TM^ SuperFrost Plus^TM^, Fisher Scientific, Loughborough, UK). Fixation and permeabilization were conducted with 4% formaldehyde/PBS for 30 min at room temperature followed by methanol/acetone (1:1) for 10 min at −20 °C. After washing thrice with PBS, the slides were preheated in a humidified chamber at 60 °C for 10 min. Preheated PNA probes (80 °C for 10 min) were then added. After 30 min, the slides were brought to room temperature and incubated for a further 2 h at room temperature in the dark. After incubation, the slides were washed twice in 2X saline-sodium citrate buffer/0.1% Tween-20/ultrapure water for 5 min each at 50 °C. After a further two washes with PBS at room temperature, blocking (Invitrogen™ eBioscience™ IHC/ICC Blocking Buffer, ThermoFisher, Oxford, UK) was performed for 30 min and labelling with CD45-Alexa Fluor 647 (1:20 dilution in blocking buffer, Biolegend, London, UK) was performed for an hour. This was followed by three washes with PBS and two washes with HBSS and staining with wheat germ agglutinin (WGA)-Alexa Fluor 488 (1 μg/mL in HBSS, ThermoFisher, Oxford, UK) and Hoechst 33342 (12 μg/mL in HBSS, ThermoFisher, Oxford, UK) for 30 min. Final washes were performed with HBSS three times and coverslips mounted (Invitrogen™ ProLong™ Diamond Antifade Mountant, ThermoFisher, Oxford, UK). The slides were allowed to cure overnight, sealed with clear nail varnish and imaged on a confocal laser scanning microscope (TCS SP8, Leica Microsystems, Milton Keynes, UK). Negative control slides were prepared with the same protocol as above but hybridized with a sense PNA probe and without staining for CD45 marker. The PNA probes that target bacterial 16S rRNA (anti-sense for positive staining: 5′-TGCCTCCCGTAGGA-3′; sense for negative staining: 5′-ACTCCTACGGGA-3′) were synthesized (Panagene, Daejeon, Republic of Korea) with the fluorophore (Alexa Fluor 594) conjugated at the N-terminus via a double linker (2-aminoethoxy-2-ethoxy acetic acid, AEEA). The probes were used at 0.5 μM prepared in an in situ hybridization buffer (Enzo Life Sciences, Exeter, UK). The acquired images were visualized in Fiji (ImageJ) software release 1.54f [31].

### 2.3. Bacterial Colony Counts of WBC and EPC Fractions Grown on Chromogenic Agar

Images of chromogenic agars cultured with NEAT, WBC and EPC fractions were cropped to a common size (1322 × 1322 pixels) and colony counts were analyzed with an open-source image processing software, ilastik (version 1.4.0) [32]. Segmentation masks were first created from the pixel classification workflow to differentiate colonies from background. This was followed by training an object classification workflow to identify individual colonies, with the classes assigned based on their species identification by MALDI-TOF. The resulting bacterial colony counts were normalized to 1000 total number of cells to reflect total abundance (CFU/1000 cells), representing all the bacteria isolated from each sample. Bacteria that were only present in the WBC fraction were deemed to be exclusively associated with white blood cells and were normalized to 1000 white blood cells (CFU/1000 WBC). The rest of the bacteria from all three fractions were considered to be associated with epithelial cells and normalized to 1000 epithelial cells (CFU/1000 EPC).

### 2.4. Bacterial DNA Extraction and 16s rRNA Sequencing

Bacterial DNA was extracted from WBC and EPC fractions separately using FastDNA^TM^ SPIN Kit for Soil (MP Biomedicals, Eschwege, Germany) in combination with mechanical lysis facilitated by Fisherbrand™ Bead Mill 24 Homogenizer (Fisher Scientific, Loughborough, UK) following the manufacturers’ instructions. Library preparation was conducted as described previously [33]. Briefly, a two-step PCR approach was used to amplify the V1V2 variable regions of the 16S rRNA gene, employing the primers 27F (3′-AGRGTTHGATYMTGGCTCAG-5′) and 338R (5′-TGCTGCCTCCCGTAGGAGT-3′). The amplification was based on the DNA abundance extracted from each sample, with the Q5^®^ High-Fidelity DNA Polymerase (New England Biolabs, Hitchin, UK) applied according to the manufacturer’s instructions.

Thermocycler settings for library preparation included an initial denaturation at 98 °C for 30 s, followed by 10 s at 98 °C, 30 s at 58 °C and 60 s at 72 °C for 15 cycles, plus another 10 cycles for barcoding and a final extension at 72 °C for 2 min. For samples with low biomass, an additional 20 cycles of one-step PCR, using the same primers, were added to enrich the 16S rRNA gene before the two-step PCR. Amplicons were assessed using the Agilent TapeStation System, with the expected size of ~450 bp. For library pooling, 10 ng of purified product from each sample quantified using the Quant-iT Picogreen dsDNA assay kit (ThermoFisher, Oxford, UK) on a microplate reader (FLUOstar Omega, BMG Labtech, Aylesbury, UK) was added. The pooled library was then purified using the QIAquick PCR Purification Kit (Qiagen, Manchester, UK) before being subjected to 2 × 300 bp paired-end Illumina MiSeq sequencing (Illumina, Cambridge, UK).

### 2.5. Sequencing Data Processing and Clustering Analysis

Sequencing data (FASTQ files) were processed using nf-core/ampliseq v2.6.1 pipeline with Nextflow v23.10.0 [34]. The pipeline is publicly available at https://github.com/nf-core/ampliseq (accessed on 22 January 2024) [35]. Briefly, sequencing quality was assessed using FastQC (v0.11.9), with results summarized and visualized through MultiQC (v1.14). Cutadapt (v3.4) was employed to trim sequences followed by DADA2 (v1.22.0) in R (v4.1.1), which removed PhiX contamination and filtered reads (before the median quality dropped below 25 and ensuring at least 90% of reads were retained). DADA2 was further utilized for the identification of Amplicon Sequence Variants (ASVs) and taxonomic classification against the SILVA database (release 138). Additionally, QIIME2 (v2022.11.1) was used for taxonomic assignment, also against the SILVA database (release 138). A total of 3179 ASVs were identified across all samples. Sequencing reads were normalized according to the formulae below to obtain theoretical pure numbers of bacteria associated with white blood cells and epithelial cells, respectively. K-means clustering of mean rank changes was performed to study bacterial community shifts between the study groups.

(1) $${Theoretical\;T}_{BN}=B_{perE}\times T_{EN}=T_{EN}\times \frac{T_{BP}\times T_{WN}-T_{WP}\times T_{BN}}{T_{EP}\times T_{WN}-T_{EN}\times T_{WP}}$$

(2) $${Theoretical\;T}_{BP}=B_{perW}\times T_{WP}=T_{WP}\times \frac{T_{BP}\times T_{EN}-T_{EP}\times T_{BN}}{T_{WP}\times T_{EN}-T_{EP}\times T_{WN}}$$

Equations (1) and (2): Normalization of sequencing data to obtain theoretical numbers of bacteria associated with white blood cells and epithelial cells. T: Total number of; B: Bacteria; W: white blood cell; E: epithelial cell; P: white blood cell fraction; N: epithelial cell fraction.

Notably, genera related to *Burkholderia* (specifically *Burkholderia-Caballeronia-Paraburkholderia*) and *Ralstonia* were excluded from the dataset prior to analysis to enhance data reliability. This decision was based on their consistent identification as contaminants in the FastDNA™ SPIN Kit for Soil (MP Biomedicals, Eschwege, Germany) [36,37,38].

### 2.6. Statistical Analyses

Prior to analysis, data distribution was checked by inspecting a histogram. If the data were abnormally distributed, log-transformation was attempted to achieve a normal distribution for parametric tests. If the data did not respond to transformation, then suitable nonparametric tests were performed. One-way ANOVA was performed on GraphPad Prism for Windows (version 10.3.1, GraphPad Software, Boston, MA, USA) to compare the three study groups for age, fresh urinary epithelial cell count (EPC/mL), fresh urinary white blood cell count (WBC/mL) and normalized bacterial abundance from agar culture (CFU/1000 cells). Two-way ANOVA was used to compare the three study groups and LUTS categories. From normalized sequencing data, relative abundances were calculated and compared between HT vs. PT and PT vs. OT using Mann–Whitney U tests. Alpha and beta diversity indices were calculated using RStudio (version 2023.06.0 Build 421, Posit Software, Boston, MA, USA). Bray–Curtis dissimilarity scores were used to evaluate differences between WBC and EPC fractions from the same sample, as well as differences within study groups using Mann–Whitney U test. K-means clustering of mean rank changes was performed and the resulting clusters were compared with one-way ANOVA using IBM SPSS Statistics for Windows (version 29.0, IBM Corp, Armonk, NY, USA). Significant differences between groups are indicated at * *p* ≤ 0.05, ** *p* ≤ 0.01 and *** *p* ≤ 0.001.

## 3. Results

### 3.1. PNA FISH and Confocal Microscopy Showed a Diversity of Bacteria Associated with Urinary White Blood Cells and Epithelial Cells

**Figure 2:**
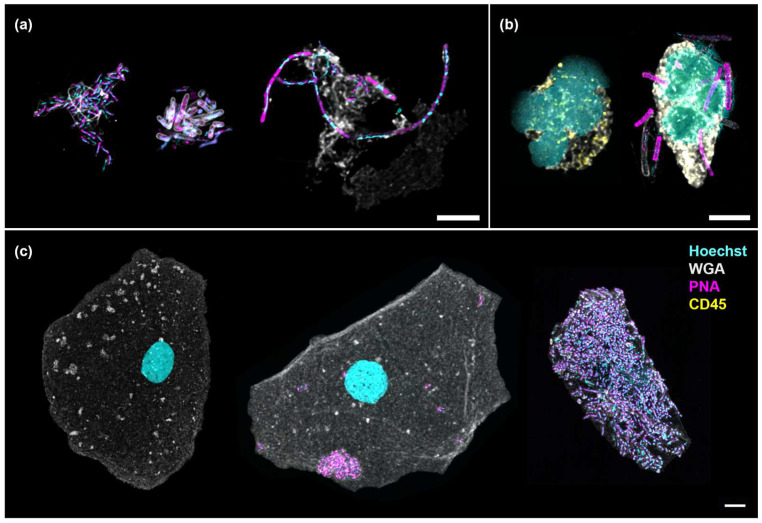
Numerous bacterial morphologies were observed in the urine, associated with or without cells. (**a**) Suspended bacterial aggregates formed by Gram-negative rods (auto-aggregates, left), a combination of Gram-negative and Gram-positive rods (co-aggregates, center) and elongated rods showing filamentation (right); (**b**) Neutrophils showing multi-lobed nucleus without (left) and with (right) associated bacteria; (**c**) An uninfected epithelial cell (left) shown in contrast with one containing auto-aggregates (center) and another enveloped by a multi-species biofilm (right). Scale bars represent 5 μm. Hoechst (cyan): DNA; WGA (gray): cytoplasm, Gram-positive bacteria and mucus; PNA (magenta): bacteria; CD45 (yellow): white blood cell.

In this study, PNA FISH and immunofluorescence techniques were employed to differentiate cellular and bacterial components. Hoechst stained the cell nuclei and bacterial DNA, whilst WGA labelled the cell cytoplasm, Gram-positive bacteria and mucus. The PNA probe tagged both Gram-positive and Gram-negative bacteria and CD45 immunofluorescence confirmed white blood cell identification. Suspended bacterial biofilms displayed structural variations, including single-species auto-aggregates, multi-species co-aggregates and filamentous forms (Figure 2a). Bacteria associated with white blood cells, as shown in Figure 2b (right), and surface-associated biofilms on epithelial cells (Figure 2c) were the objects of interest in this study. These initial microscopy observations validated the approach of isolating WBC- and EPC-associated bacteria to examine immune targets in chronic UTI.

### 3.2. The Three Study Groups Were Distinguished by Urinary White Blood Cell Counts and Lower Urinary Tract Symptoms

**Figure 3:**
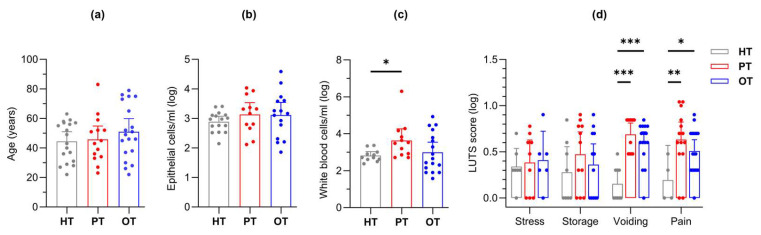
Study cohort (HT, PT and OT) characteristics. The groups were characterized by (**a**) age, fresh microscopy counts of urinary (**b**) epithelial and (**c**) white blood cells; (**d**) LUTS scores were categorized into stress, storage, voiding and pain. The PT group showed significantly elevated levels of white blood cell count as well as voiding and pain scores. Bar charts show the mean with 95% CI. Significant differences between groups are indicated at * *p* ≤ 0.05, ** *p* ≤ 0.01 and *** *p* ≤ 0.001 (one-way (**a**–**c**) or two-way (**d**) ANOVA with Fisher’s LSD test for multiple comparisons). HT: healthy; PT: chronic UTI pre-treatment; OT: chronic UTI on treatment; LUTS: lower urinary tract symptoms.

This study modelled disease progression across healthy individuals (HT), chronic UTI patients yet to receive treatment (PT) and chronic UTI patients undergoing treatment (OT). Participant ages were similar (mean years [95% CI]: HT = 44.4 [37.8, 51.0]; PT = 45.7 [36.5, 54.9]; OT = 50.9 [42.0, 59.8]) (Figure 3a), as were epithelial cell counts (mean cells/mL [95% CI]: HT = 893 [522, 1263]; PT = 2588 [557, 4618]; OT = 4009 [−413.2, 8432]) (Figure 3b); however, fresh urinary white blood cell counts were significantly elevated in PT (mean cells/mL [95% CI]: HT = 528 [175, 882]; PT = 146,315 [−160,573, 453,202]; OT = 9254 [−758, 19,265]) (Figure 3c). Using a 39-point LUTS questionnaire, stress and storage symptoms showed no significant differences between the study groups but chronic UTI patients in both PT and OT scored consistently higher than HT in voiding (mean score [95% CI]: HT = 0.88 [0.36, 1.39]; PT = 4.14 [2.50, 5.79]; OT = 3.68 [2.59, 4.77]) and pain (mean score [95% CI]: HT = 0.44 [−0.04, 0.91]; PT = 5.36 [3.44, 7.27]; OT = 3.53 [2.52, 4.53]) symptoms (Figure 3d). Both urinary WBC counts and LUTS scores were reduced in the OT group when compared to the PT group.

### 3.3. Gold Standard Diagnostic Tests for UTI Were Unable to Reliably Distinguish Between the Three Study Groups

Positive leukocyte esterase and nitrite tests are commonly used as indicators for UTI. Within HT, biochemical dipstick results were not available for five samples; however, 2/13 (15%) samples tested positive for leukocyte esterase, while 5/14 (36%) in PT and 8/19 (42%) in OT were positive. None of the samples in HT were positive for nitrite, compared to 3/13 (23%) in PT and 2/19 (11%) in OT. For MSU culture, the hospital laboratory reports samples as “MSU-positive” when there is growth of >10^5^ CFU/mL of a single known uropathogen. “MSU-negative with no significant growth” is reported when growth is below the aforementioned threshold, not a “known” uropathogen, or growth is absent altogether. “MSU-negative with mixed growth” is reported when two or more types of bacterial colonies are observed. With these interpretation criteria, all 18 HT samples were MSU-negative with no significant growth, although colonies were actually observed in 56% of the samples. In the PT group, 5/14 (36%) were MSU-positive, showing either *Escherichia coli* or Coliforms (*Klebsiella/Enterobacter/Serratia* group); the remaining 64% were MSU-negative with either no significant growth or mixed growth. In the OT group, 3/19 (16%) were MSU-positive, with *E. coli*, *Klebsiella pneumoniae* and *Citrobacter braakii* identified. The remaining OT samples (84%) were MSU-negative with either no significant growth or mixed growth. Both dipstick and MSU tests exhibited low sensitivity, averaging below 30%, in detecting UTI in the patient groups (PT and OT).

### 3.4. Enriched Urine Culture Following CD45 Sorting into WBC and EPC Fractions Uncovered Bacteria Uniquely Associated with White Blood Cell and Epithelial Cell in All Study Groups

**Figure 4:**
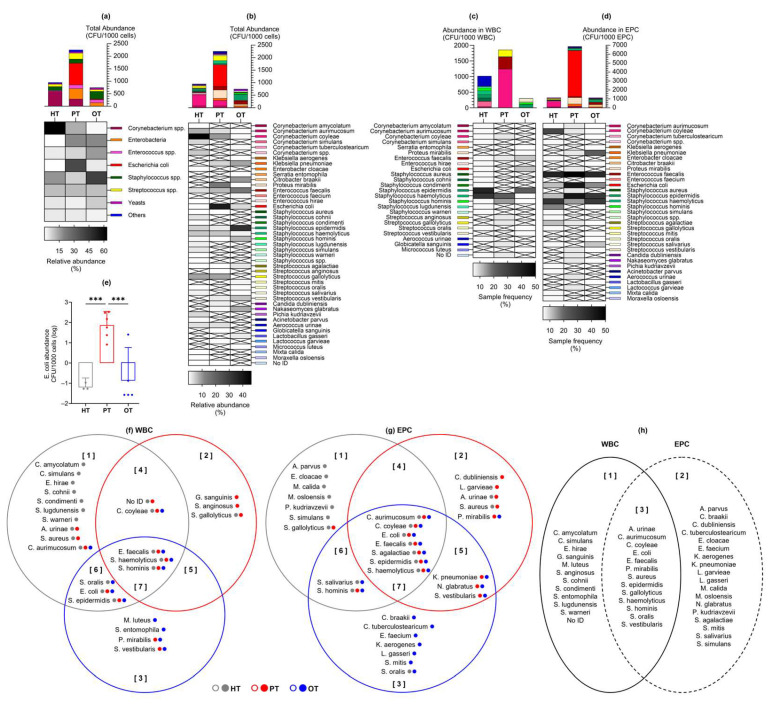
Abundance of bacteria and yeast isolated from urine culture following CD45 sorting into WBC and EPC fractions. Total abundance (CFU/1000 cells) is shown in (**a**) microbial groups or (**b**) individual species, as well as those associated with (**c**) WBC and (**d**) EPC. Bar charts show the mean with 95% CI while heatmaps show relative bacterial abundance (**a**,**b**) and sample frequency (**c**,**d**). There were no significant differences in the mean CFU/1000 cells between study groups, except for (**e**) *Escherichia coli* at *** *p* ≤ 0.001 (one-way ANOVA with Fisher’s LSD test for multiple comparisons). The Venn diagrams show bacteria species that were associated with (**f**) WBC and (**g**) EPC in the three study groups. The colored dots after each species name refer to their presence in the study groups. For instance, in diagram (**f**) set [1], *Corynebacterium aurimucosum* was isolated from all study groups (gray, red and blue dots) but only associated with WBC in HT (gray circle). The diagram in (**h**) shows an overview of the species found only in WBC [1], EPC [2] or both fractions [3]. HT: healthy; PT: chronic UTI pre-treatment; OT: chronic UTI on treatment; WBC: white blood cell fraction; EPC: epithelial cell fraction; CFU: colony-forming unit.

Laboratory culture on chromogenic agar yielded colony growth in 17/18 (94%), 14/14 (100%) and 18/19 (95%) of HT, PT and OT samples, respectively. A total of 41 bacteria and 3 yeast species from 21 genera, 15 families, 7 orders, 4 classes and 4 phyla was identified (Table S1). Total abundance was the highest in PT, followed by HT and OT (mean CFU/1000 cells [95% CI]: HT = 52.9 [14.5, 91.3]; PT = 161.3 [28.8, 293.7]; OT = 39.4 [7.7, 71.0]) (Figure 4a). Based on the relative abundance shown in the heatmap in Figure 4a, from HT to PT and PT to OT, notable trends included a decline in *Corynebacterium* spp. while Enterobacteria and *Enterococcus* spp. increased. *Escherichia coli* showed the highest abundance in PT and was significantly reduced in OT (mean CFU/1000 cells [95% CI] in HT = 0.013 [−0.003, 0.028]; PT = 61.910 [−4.263, 128.100]; OT = 1.415 [−1.544, 4.374]) (Figure 4e). *Staphylococcus* spp. increased in OT and yeasts were also slightly more common in OT. *Streptococcus* spp. and the bacterial group ‘Others’ remained relatively stable among the study groups. There were 30, 21 and 23 species isolated from HT, PT and OT, respectively, giving the highest species richness in individuals without UTI. The most abundant species was *Corynebacterium coyleae* in HT, *E. coli* in PT and *Staphylococcus epidermidis* in OT (Figure 4b). Both alpha- and beta-diversity analyses did not show apparent differences between the study groups.

By sorting the urinary cells with CD45 marker prior to laboratory culture, bacteria exclusively associated with WBC were highlighted as potential immune targets and infective agents (Figure 4c). The species that was most frequently associated with WBC was *S. epidermidis* in HT and OT, and *Staphylococcus haemolyticus* in PT. Bacteria that were associated with WBC in all study groups were *Enterococcus faecalis*, *S. haemolyticus* and *Staphylococcus hominis*. Most bacteria associated with WBC in HT were found only in this study group (Figure 4f set 1). In PT, bacteria that were only present in this study group and associated with WBC were *Streptococcus anginosus* and *Globicatella sanguinis* (Figure 4f set 2). *Serratia entomophila* and *Micrococcus luteus* were associated with WBC solely in OT (Figure 4f set 3). No yeasts were associated with WBC in any of the study groups.

Bacteria associated with epithelial cells in the EPC fraction (Figure 4d) were postulated to be bladder-resident commensals or pathogens that were shed along with the cells. Species that were isolated from EPC in all three study groups were *Corynebacterium aurimucosum*, *C. coyleae*, *E. coli*, *Streptococcus agalactiae*, *S. epidermidis* and *S. haemolyticus*, with *E. faecalis* being the most prevalent. Most EPC-associated bacteria in HT and OT were distinctively found in these study groups (Figure 4g sets 1 and 3), while the species found in PT frequently showed overlaps with HT and OT. This demonstrated a plausible shift in bacterial communities in the bladder during health and chronic UTI.

### 3.5. 16S rRNA Sequencing Showed Lactobacillus as the Predominant Genus Across All Study Groups and Differences Between WBC and EPC Fractions

The microbial ecology of the study groups was further analyzed using 16S rRNA sequencing, with each urine sample contributing two fractions (WBC and EPC) after CD45-sorting, thus amounting to 100 sequencing samples from 17 HT (one sample was lost during processing), 14 PT and 19 OT individuals. All the samples yielded sufficient sequencing reads for analysis. A total of 3179 amplicon sequencing variants (ASVs) was classified across 25 phyla, 144 classes, 106 orders, 172 families and 476 genera (Table S2).

**Figure 5:**
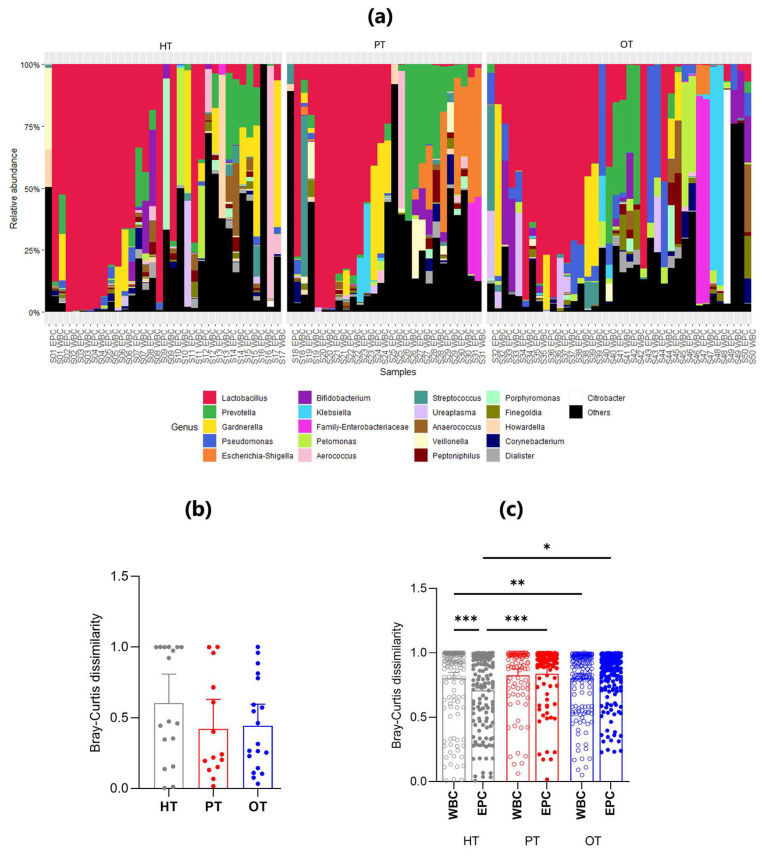
The relative abundance and mean Bray-Curtis dissimilarity scores between WBC and EPC fractions in 16S rRNA sequencing. (**a**) Stacked bar charts show the relative abundance of the top 21 genera with *Lactobacillus* being the predominant genus. Within each study group (HT, PT and OT), the WBC and EPC fractions from the same sample are shown side by side; (**b**) Nested Bray-Curtis dissimilarity score between WBC and EPC microbial ecology within an individual in the respective study groups; (**c**) Bray–Curtis dissimilarity scores between WBC and EPC bacteria. Bar charts show the mean with 95% CI. Significant differences between groups are indicated at * *p* ≤ 0.05, ** *p* ≤ 0.01 and *** *p* ≤ 0.001 (Mann–Whitney U test). HT: healthy; PT: chronic UTI pre-treatment; OT: chronic UTI on treatment; WBC: white blood cell fraction; EPC: epithelial cell fraction.

There were 21 genera that accounted for more than 85% of the sequencing reads across all study groups (Figure 5a) and *Lactobacillus* was the most abundant genus, dominating 15/34 (44.1%) HT, 9/28 (32.1%) PT and 10/38 (26.3%) OT samples. *Lactobacillus* presence was also consistent in the WBC-associated microbiomes across study groups, in 7/17 (41.2%) HT, 5/14 (35.7%) PT and 5/19 (26.3%) OT samples. These findings suggested that *Lactobacillus* dominated the bladder microbial ecology with the immune system targeting *Lactobacillus* in both healthy and patient groups.

There was increased similarity between WBC- and EPC-associated bacteria in both the PT and OT compared with HT groups, a finding supported by the nested Bray-Curtis dissimilarity test (Figure 5b). The nested mean dissimilarity score was the highest in HT (0.605), compared to 0.422 and 0.443 in PT and OT, respectively, though these differences were not statistically significant. This observation was further confirmed by the level of dissimilarity between WBC-associated and EPC-associated bacteria across all study groups (Figure 5c). In HT, WBC-associated bacteria exhibited a significantly higher dissimilarity score than EPC-associated bacteria. Non-significant differences were observed in PT and OT.

Among WBC-associated bacteria, the only statistically significant difference was noted between HT and OT, indicating that white blood cells may target distinct bacterial groups across individuals, regardless of health status (Figure 5c). Conversely, the dissimilarity of EPC-associated microbiomes significantly increased from HT to both PT and OT, suggesting that the epithelium-associated microbiome gradually became more heterogeneous.

### 3.6. Bacterial Signatures in the Urine Were Related to Lower Urinary Tract Symptoms in Chronic UTI

**Figure 6:**
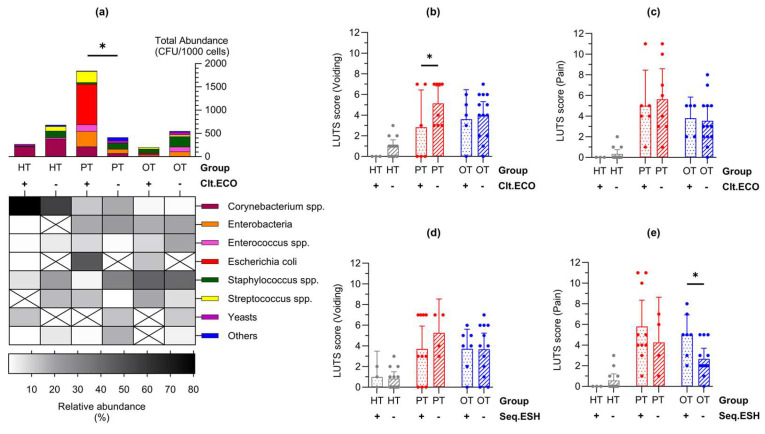
Lower urinary tract symptoms differed in individuals with *Escherichia coli* in enriched culture and *Escherichia-Shigella* group in 16S rRNA sequencing. (**a**) Abundance bar charts are shown in microbial groups with their relative abundance shown in the heatmap for samples with or without *E. coli* growth in culture (Clt.ECO). Total abundance was higher in PT samples containing *E. coli*; (**b**,**c**) Voiding symptom score was higher in samples that were Clt.ECO- in PT while there were no differences in the pain score; (**d**,**e**) Conversely, there were no differences in the voiding symptom score but pain score was higher in samples containing *Escherichia-Shigella* group in sequencing (Seq.ESH). Bar charts show the mean with 95% CI. Significant differences between groups are indicated at * *p* ≤ 0.05 (two-way ANOVA with Fisher’s LSD test for multiple comparisons). HT: healthy; PT: chronic UTI pre-treatment; OT: chronic UTI on treatment; CFU: colony-forming unit.

In enriched urine culture following CD45 sorting into WBC and EPC fractions, only *E. coli* showed statistically significant differences across study groups (Figure 4e). Consequently, samples were categorized based on the presence or absence of *E. coli* growth in culture (indicated as “Clt.ECO+” or “Clt.ECO−”) for analysis. *Corynebacterium* spp., *Enterococcus* spp. and *Staphylococcus* spp. were consistently present in all study groups (Figure 6a); however, *Streptococcus* spp., Enterobacteria and “Others” were absent in HT^Clt.ECO+^, HT^Clt.ECO−^ and OT^Clt.ECO+^, respectively. Yeasts were found in HT^Clt.ECO+^ but were absent in PT^Clt.ECO+^ and OT^Clt.ECO+^. Samples containing *E. coli* showed a higher total abundance than those without, reaching statistical significance within PT (mean CFU/1000 cells [95% CI]: HT^Clt.ECO+^ = 90.0 [−173.8, 353.8], n = 3; HT^Clt.ECO−^ = 48.7 [5.9, 91.6], n = 14; PT^Clt.ECO+^ = 307.4 [1.2, 613.6], n = 6; PT^Clt.ECO−^ = 51.6 [−6.4, 109.7], n = 8; OT^Clt.ECO+^ = 40.5 [−5.3, 86.3], n = 5; OT^Clt.ECO−^ = 42.0 [−4.4, 88.3], n = 13). When matched to the individual’s symptoms (Figure 6b,c), voiding scores were generally higher in samples without *E. coli*, showing statistical significance in PT (mean score [95% CI]: HT^Clt.ECO+^ = 0.00 [0.00, 0.00]; HT^Clt.ECO−^ = 1.00 [0.39, 1.61]; PT^Clt.ECO+^ = 2.83 [−0.77, 6.43]; PT^Clt.ECO−^ = 5.13 [3.43, 6.82]; OT^Clt.ECO+^ = 3.60 [0.74, 6.46]; OT^Clt.ECO−^ = 4.00 [2.70, 5.31]).

A similar analysis was conducted on *E. coli* presence identified via 16S rRNA sequencing, broadly represented by the *Escherichia-Shigella* group (indicated as “Seq.ESH+” or “Seq.ESH−”). The voiding symptom scores showed a similar trend (Figure 6d) and pain scores were significantly higher in OT samples containing *Escherichia-Shigella* group in sequencing (mean score [95% CI] in HT^Seq.ESH+^ = 0.00 [0.00, 0.00], n = 4; HT^Seq.ESH−^ = 0.58 [−0.05, 1.22], n = 13; PT^Seq.ESH+^ = 5.80 [3.26, 8.34], n = 10; PT^Seq.ESH−^ = 4.25 [−0.13, 8.63], n = 4; OT^Seq.ESH+^ = 5.00 [3.08, 6.93], n = 7; OT^Seq.ESH−^ = 2.67 [1.64, 3.69], n = 12) (Figure 6e). Additional clinical signs and symptoms related to the presence of *E. coli* in culture or the *Escherichia-Shigella* group in sequencing are detailed in Figure S1.

### 3.7. Clustering Analysis of Mean Rank Changes in Relative Bacterial Abundance Revealed Significant Ecological Shifts Amidst a Stable Bacterial Community Between the Study Groups

**Figure 7:**
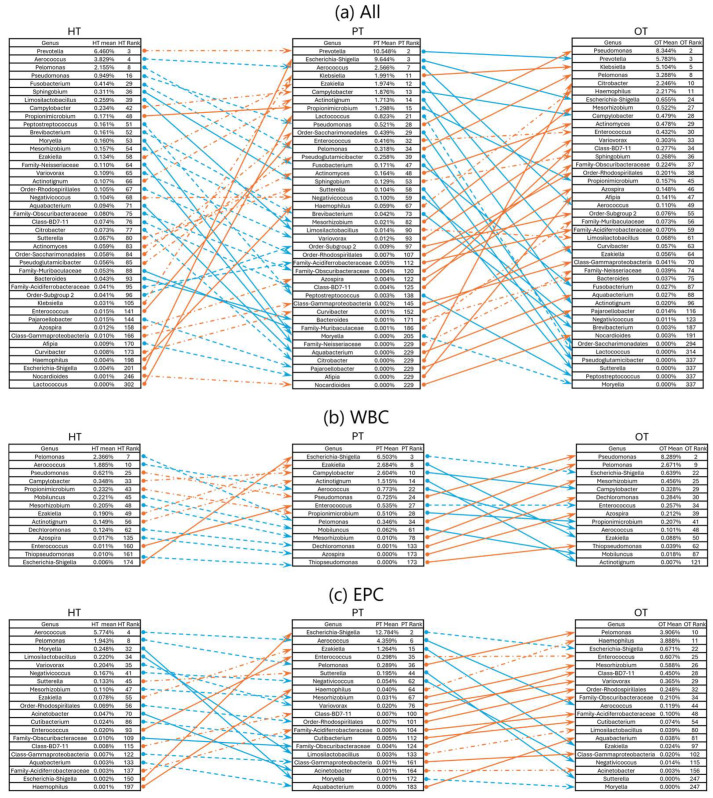
Rank changes in relative bacterial abundance from HT to PT and PT to OT in 16S rRNA sequencing. Lists of genera showing an increase (orange lines) and decrease (blue lines) in ranks based on their mean relative abundance in the respective study groups in (**a**) all samples; (**b**) WBC and (**c**) EPC. Dashed lines show no significant difference while significant differences between the study groups are indicated with solid lines at *p* ≤ 0.05 (Mann–Whitney U). HT: healthy; PT: chronic UTI pre-treatment; OT: chronic UTI on treatment; WBC: white blood cell fraction; EPC: epithelial cell fraction.

To examine how the bacterial ecology shifted from the healthy population to diseased, two pairs of study group comparisons were conducted—from HT to PT and PT to OT. When analyzing the microbial ecologies associated with WBC and EPC collectively, 42 genera exhibited significant shifts in relative abundance. Of these, 12 genera showed significant changes between HT and PT, while 33 genera differed between PT and OT (Figure 7a). Specifically, WBC-associated bacteria revealed changes in 2 genera from HT to PT and 12 genera from PT to OT (Figure 7b). EPC-associated bacteria displayed significant alterations in 7 genera from HT to PT and 14 genera from PT to OT (Figure 7c). There were no significant differences in the alpha- and beta-diversity indices in the sequenced samples within and between the study groups.

**Figure 8:**
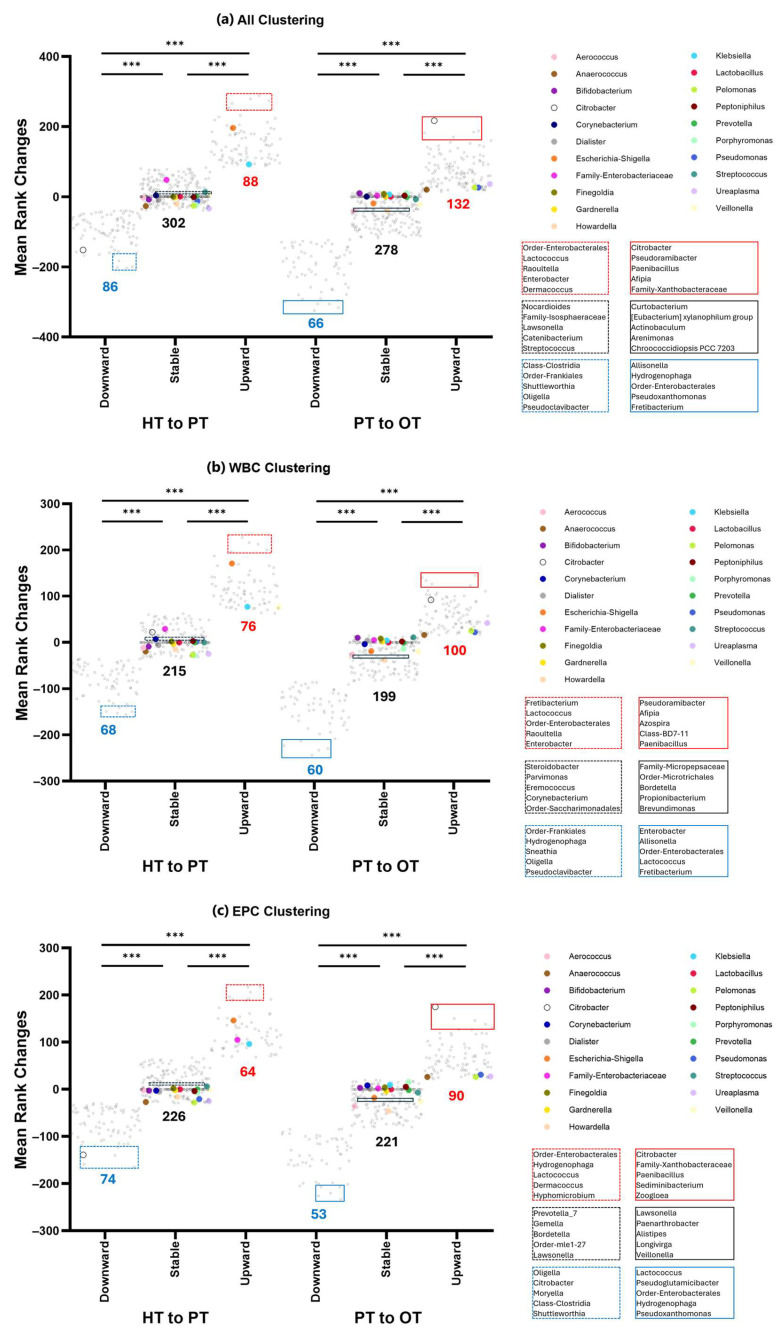
K-means clustering of 16S rRNA sequencing data showed three distinct clusters that shifted from the healthy population to diseased. The number of genera that comprises downward, stable or upward are indicated below the clusters shown in (**a**) all samples; (**b**) WBC and (**c**) EPC. Genera with the highest relative abundances (top 21) are color-coded and mostly belonged in the stable communities. Bacteria that showed the least amount of rank changes in the stable clusters around y = 0 are highlighted (black) and listed. The top 5 genera with the largest upward (red) or downward (blue) shifts are also highlighted and listed. Within each comparison from HT to PT (dashed lines) and PT to OT (solid lines), the three clusters are significantly different from one another at *** *p* ≤ 0.001 (one-way ANOVA with Bonferroni test for multiple comparisons). HT: healthy; PT: chronic UTI pre-treatment; OT: chronic UTI on treatment; WBC: white blood cell fraction; EPC: epithelial cell fraction.

To capture broader bacterial changes across the healthy population and diseased states, K-means clustering was applied to categorize shifts of all 476 genera into three patterns—upward shifts, stable bacterial communities and downward shifts. This analysis was conducted on all samples (Figure 8a) as well as within WBC (Figure 8b) and EPC (Figure 8c) fractions. The majority of the top 21 abundant genera (61.9%) remained stable across all study groups. Exceptions included *Escherichia-Shigella*, *Klebsiella*, *Veillonella* and *Family-Enterobacteriaceae*, which increased from HT to PT in both WBC and EPC but showed no significant changes from PT to OT. Notably, *Anaerococcus*, *Pelomonas*, *Pseudomonas* and *Ureaplasma* consistently showed a slight upward shift from PT to OT across WBC and EPC fractions. In contrast, EPC-associated *Citrobacter* decreased from HT to PT but bloomed in both WBC and EPC from PT to OT, suggesting a potentially beneficial role in health by regulating this genus. When comparing bacteria identified in the upward shift clusters with our culture data, previous studies [39,40,41,42] and manufacturers’ manuals (https://www.biomerieux-usa.com/sites/subsidiary_us/files/18_chromid_cpse_flyer.pdf; https://www.biomerieux-nordic.com/sites/subsidiary_no/files/9308627-002-gb-a-chromid-cps-elite-reading-guide.pdf, accessed on 20 January 2025), only 4 to 12% were reported to grow on chromogenic agar. Full bacterial listings for each cluster are provided in Table S3.

## 4. Discussion

This is the first study to investigate urinary microbiota associated with an immune response in chronic UTI. Congruent with previous works, bacteria identified using agar culture and 16S rRNA sequencing in this study were predominantly Firmicutes, Proteobacteria, Actinobacteria and Bacteroidetes [30]. Although the culture was limited to chromogenic agar and overnight aerobic incubation at 37 °C, only 2/51 (3.9%) samples showed an absence of growth, and 41 bacterial species were identified in this cell-enriched method. In sequencing, all samples were sequence-positive and 476 genera were identified, representing a 20 to 40% increase over previous reports [7,21]. Many previous urobiome studies utilized a small volume of urine collected via transurethral catheterization and DNA extraction by enzymatic lysis for 16S rRNA sequencing [21,24,27,43,44]. The broader diversity achieved in this study may be attributed to bacterial tropism toward cells in the urine [10,15,19,45,46,47,48], use of mechanical lysis during DNA extraction and sequencing of different hypervariable regions of the 16S rRNA gene.

We posited that bacteria associated with WBC were pathogens that were targets of the host immune system while bacteria associated with EPC were a rich ecology made up of commensals and pathogens. Bacterial colonies that were WBC-exclusive in culture were most frequently observed in HT samples (16/18, 88.9%), followed by PT (9/14, 64.3%) and OT (10/19, 52.6%). Similarly, in 16S rRNA sequencing, differences between both fractions were the most prominent in HT compared to chronic UTI patients (PT and OT) (Figure 5b). Urothelium acts as a protective barrier and epithelial cell sloughing is a known bladder immune mechanism that helps to reduce bacterial load in UTI [10,12,16,46]. In this study, the number of fresh urinary EPC/mL was similar in all cohorts (Figure 3b). However, we did not inspect the epithelial cell in all individuals to determine the extent of bacterial association. It is quite possible that the number of epithelial cells colonized by bacteria is notably higher in chronic UTI [10]. Indeed, the bacterial load associated with EPC fraction in PT was remarkably higher than controls in this study (Figure 4d). The presence of WBC-exclusive bacteria in HT suggested transient subclinical infections but, in healthy individuals, the bladder epithelial barrier is likely to be intact with rigorous immunosurveillance by white blood cells. On the contrary, the urothelial structural integrity may have been compromised in chronic UTI patients giving way to colonizing bacteria, thus leading to a lesser degree of cellular compartmentalization which was reflected in the diminishing distinction and increasing species overlap between WBC and EPC fractions. Some bacteria were more frequently associated with WBC than others, but WBC-exclusive species, or genera, remained discrete within and between study groups.

Unlike previous studies employing similar culture conditions [7,19], only one species of *Lactobacillus* (*L. gasseri*) was isolated from one OT sample in this study. Nevertheless, *Lactobacillus* was the most abundant genera in sequencing across all study groups, consistent with prior urobiome studies in women [21,24,27,43,44]. *Lactobacilli* are facultative anaerobic Gram-positive bacteria from the phylum Firmicutes and form part of the microbiota in human gastrointestinal, urinary and female genital tracts. They are generally regarded as beneficial bacteria with protective functions, such as enhancing epithelial barrier function, promoting mucus production, colonization resistance through competition and immunomodulation via their metabolites, or by inducing production of antibodies and antimicrobial peptides [49,50]. However, studies at the species-level hinted at different biology shaped by different *Lactobacillus* species. For instance, *L. crispatus* was associated with a stable and normal vaginal microflora while *L. gasseri* and/or *L. iners* increased the risk of vaginal dysbiosis during pregnancy [51]. In the bladder, *L. crispatus* was commonly found in health while *L. gasseri* was detected more frequently in individuals with urgency urinary incontinence (UUI) [21]. In our study, we found that *Lactobacillus* was associated with both WBC and EPC fractions in all study groups, suggesting that defined populations of this bacterial group were actively targeted by the immune system. Future work that aims to characterize *Lactobacillus* down to the species or strain levels may be able to shed light on the regulatory roles exhibited by this genus in the bladder microbiome.

By characterizing samples based on the presence of *E. coli* in culture or *Escherichia-Shigella* group in sequencing, we established the occurrence of a bacterial fingerprint that affected the overall urobiome composition, which was also relevant to the symptoms experienced. Chronic UTI patients (PT) that grew *E. coli* in culture scored lower in voiding symptoms while those (OT) that showed *Escherichia-Shigella* in sequencing reported more severe pain (Figure 6). Both symptom categories have been described as the most indicative of pyuria and chronic UTI [13]. The prevalence of *E. coli* and *Escherichia-Shigella* was relatively low in this study, and it is important to clarify that they were not identified as the cause of infection. Instead, this bacterial group was used as a guide species to indicate urobiome shifts and stratify the study cohorts. Such classification may inform the severity of infection and benefit patients by tailoring a more efficient approach in disease-monitoring and treatment.

Past studies have also attempted to arrange samples into urotypes based on bacterial diversity or predominant bacterial groups but the resulting clusters did not differentiate the healthy individuals from those with UUI studied [21,24,25]. Another study grouped urobiomes based on the relative abundance of sequenced *Lactobacillus* and found that UUI in individuals with mixed urinary incontinence showed bacterial communities characterized by low *Lactobacilli* and high Proteobacteria [43]. In this study, the application of clustering analysis to mean rank changes revealed group trends in bacterial shifts that could describe health and different stages of disease as well as microbial responses to treatment (Figure 8). The varying bacterial community shifts from HT to PT and PT to OT may prompt a shift from the strongly-held belief that refractory UTIs are monomicrobial and only caused by specific bacteria. Instead, polymicrobial infection is highly probable in chronic UTI, as has recently been described in catheter-associated UTI [52].

This study is the first to investigate immune system-associated bacteria in chronic UTI, providing novel insights into host–microbe interactions. By integrating traditional culture-based methods with 16S rRNA sequencing, it offers a comprehensive comparison of bacterial identification techniques. Moreover, sampling from well characterized chronic UTI cohorts, particularly a pre-treatment cohort, adds considerable robustness to our findings. Nonetheless, the study is limited by its sample size, highlighting the need for a larger cohort to enhance statistical power. Future research should also explore differences in immune responses and bacterial composition between pre- and postmenopausal patients, as well as between healthy controls in these groups. Furthermore, as bacterial identification was limited to the genus level due to technical constraints in sequencing, species-level resolution would offer deeper insights into microbial dynamics in chronic UTI.

## 5. Conclusions

In our study, bacteria uniquely associated with WBC or EPC were uncovered. However, likely due to inter-person urobiome variances, these distinctions were not absolute. Nonetheless, we were able to discern signature changes in bacterial communities within each individual as the central distinguishing feature in chronic UTI. This reinforced the lack of a universal commensal/pathogen divide based on a few specific species or abundance thresholds as are commonly used in standard diagnostic tests. Although it may be difficult to determine the reason for such dysbiosis in chronic UTI, further examination into the consequent metabolomic and functional changes are warranted [53,54]. For future studies, the addition of a study group comprising individuals that have completely recovered or longitudinal studies tracing chronic UTI patients are needed. Studying individuals through their treatment period until partial or complete remission could determine whether a return to health constitutes a complete transformation or simply rehabilitation of the urobiome to pre-disease state.

## Data Availability

The original contributions presented in this study are included in the article and Supplementary Materials. Further inquiries can be directed to the corresponding author.

## Supplementary Materials

The following supporting information can be downloaded at: https://www.mdpi.com/article/10.3390/pathogens14030299/s1, Figure S1: Clinical signs and symptoms with presence or absence of *E. coli* growth in culture and *Escherichia-Shigella* group in 16S rRNA sequencing following CD45 sorting of urinary cells; Table S1: List of microbial species identified from culture of WBC and EPC fractions on chromogenic agar (grouped); Table S2: List of all genera identified in 16S rRNA sequencing; Table S3: List of all genera in the different clusters shown in Figure 8.
