# Supplementary material for: Novel Techniques to Unravel Causative Bacterial Ecological Shifts in Chronic Urinary Tract Infection
Source: Pathogens. 2025 Mar 20;14(3):299. doi: 10.3390/pathogens14030299 (PMC11944610; doi:10.3390/pathogens14030299)
Supplement: Supplementary file 1 [file pathogens-14-00299-s001.zip › Figure S1.pdf]

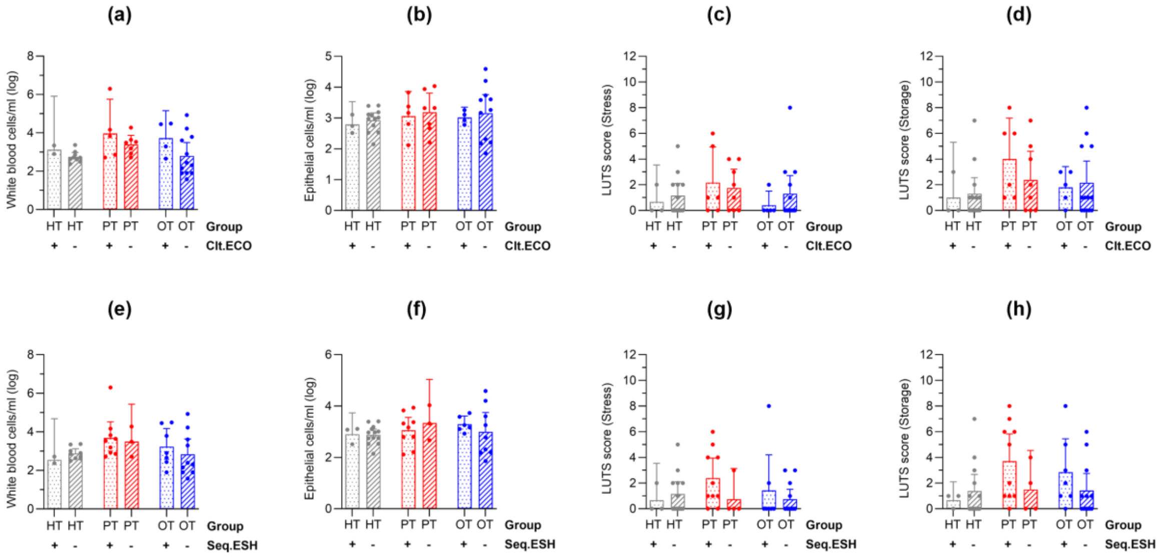

Figure S1. Clinical signs and symptoms of samples with presence (+) or absence (-) of *E. coli* growth in culture (a-d) and *Escherichia-Shigella* group in 16S rRNA sequencing (e-h) following CD45 sorting of urinary cells. HT: healthy; PT: chronic UTI pre-treatment; OT: chronic UTI on treatment; Clt.ECO: *E. coli* in culture; Seq.ESH: *Escherichia-Shigella* group in 16S rRNA sequencing.
