# Supplementary material for: Novel Techniques to Unravel Causative Bacterial Ecological Shifts in Chronic Urinary Tract Infection
Source: Pathogens. 2025 Mar 20;14(3):299. doi: 10.3390/pathogens14030299 (PMC11944610; doi:10.3390/pathogens14030299)
Supplement: Supplementary file 1 [file pathogens-14-00299-s001.zip › Table S1.pdf]

Table S1. List of microbial species identified from culture of WBC and EPC fractions on chromogenic agar (grouped).

***Corynebacterium* spp.**	***Staphylococcus* spp.**
Corynebacterium amycolatum<br>*Corynebacterium aurimucosum*<br>*Corynebacterium coyleae*<br>*Corynebacterium simulans*<br>*Corynebacterium tuberculostearicum*<br>*Corynebacterium* spp.	*Staphylococcus aureus*<br>*Staphylococcus cohnii*<br>*Staphylococcus condimenti*<br>*Staphylococcus epidermidis*<br>*Staphylococcus haemolyticus*<br>*Staphylococcus hominis*<br>*Staphylococcus lugdunensis*<br>*Staphylococcus simulans*<br>*Staphylococcus warneri*<br>*Staphylococcus spp.*
**Enterobacteria**	***Streptococcus* spp.**
Klebsiella aerogenes<br>*Klebsiella pneumoniae*<br>*Enterobacter cloacae*<br>*Serratia entomophila*<br>*Citrobacter braakii*<br>*Proteus mirabilis*	*Streptococcus agalactiae*<br>*Streptococcus anginosus*<br>*Streptococcus gallolyticus*<br>*Streptococcus mitis*<br>*Streptococcus oralis*<br>*Streptococcus salivarius*<br>*Streptococcus vestibularis*
***Enterococcus* spp.**	**Yeasts**
Enterococcus faecalis<br>*Enterococcus faecium*<br>*Enterococcus hirae*	*Candida dubliniensis*<br>*Nakaseomyces glabratus*<br>*Pichia kudriavzevii*
Escherichia coli	**Others**
Escherichia coli	*Acinetobacter parvus*<br>*Aerococcus urinae*<br>*Globicatella sanguinis*<br>*Lactobacillus gasseri*<br>*Lactococcus garvieae*<br>*Micrococcus luteus*<br>*Mixta calida*<br>*Moraxella osloensis*<br>No ID
